# Dracunculiasis in South Sudan

**DOI:** 10.4269/ajtmh.2010.09-0681

**Published:** 2010-05

**Authors:** Christian Fabiansen, Zitta Barrella Harboe, Vibeke Christensen

**Affiliations:** Médecins Sans Frontières-Denmark, Kristianiagade 8, DK-2100 København Ø, Denmark

A Dinka-tribesman came to Marial Lou Hospital, South Sudan, in 2005.

**Figure 1.**
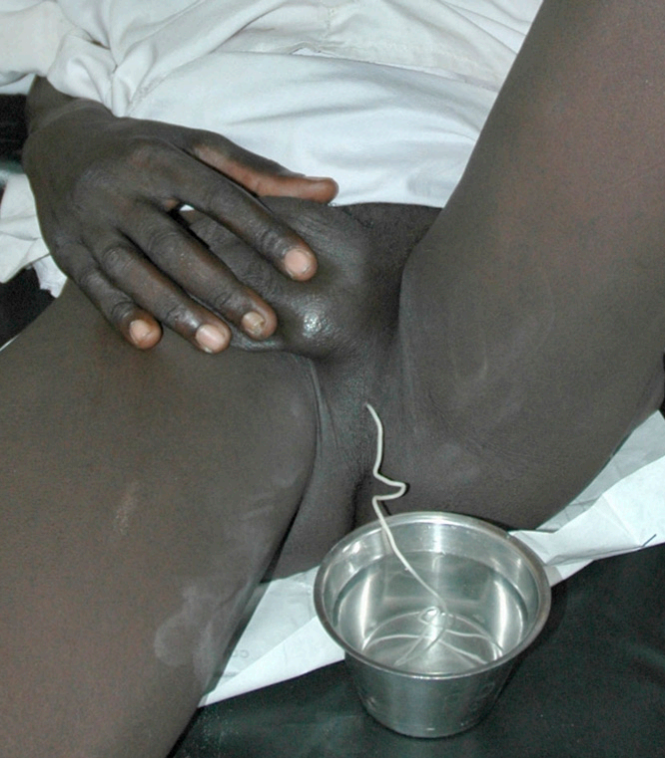
Dracunculus Medinensis emerging from patient's perineum. This figure appears in color at www.ajtmh.org.

**Figure 2.**
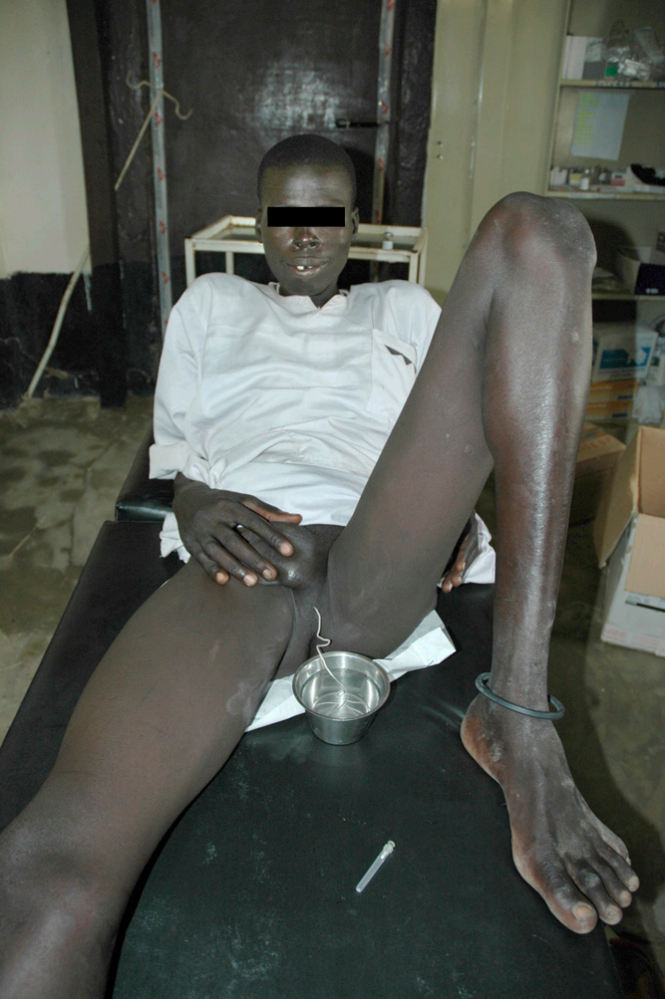
Dracunculus Medinensis emerging from patient's perineum. This figure appears in color at www.ajtmh.org.

He experienced severe discomfort from a Dracunculus Medinensis (Guinea worm) emerging from his perineum (see Figures 1 and 2). The 80-cm-long parasite was successfully extracted.

Persons get Dracunculiasis by drinking water containing water fleas harboring the larvae of the worm. The larvae are released into the water by the adult worm that emerges through the skin of infected people.

Eighty percent of cases today exist in South Sudan. Sudan's civil war officially ended in 2005 after decades of fighting. Following the peace agreement the Southern Sudan Guinea Worm Eradication Program was created in 2006, counting thousands of village volunteers and health staff. The same year an increase of 270% (> 20,000 cases) occurred because of prior underreporting.^1^ In the first 6 months of 2009 only 1,188 cases were reported. New episodes of violence in South Sudan pose the greatest challenge for again giving Dracunculiasis the upper hand. In the first half of 2009, 23 incidents of insecurity were reported to disrupt program operations.^2^ Widespread violence will make coherent surveillance and provision of safe drinking water impossible leaving sporadic treatment to medical humanitarian organizations. Keeping peace contains the hope not only of preventing cases but altogether eradicating Dracunculiasis.
